# Conformational B-Cell Epitopes Prediction from Sequences Using Cost-Sensitive Ensemble Classifiers and Spatial Clustering

**DOI:** 10.1155/2014/689219

**Published:** 2014-06-17

**Authors:** Jian Zhang, Xiaowei Zhao, Pingping Sun, Bo Gao, Zhiqiang Ma

**Affiliations:** ^1^School of Computer Science and Information Technology, Northeast Normal University, Changchun 1300117, China; ^2^The Engineering Laboratory for Drug-Gene and Protein Screening, Northeast Normal University, Changchun 1300117, China

## Abstract

B-cell epitopes are regions of the antigen surface which can be recognized by certain antibodies and elicit the immune response. Identification of epitopes for a given antigen chain finds vital applications in vaccine and drug research. Experimental prediction of B-cell epitopes is time-consuming and resource intensive, which may benefit from the computational approaches to identify B-cell epitopes. In this paper, a novel cost-sensitive ensemble algorithm is proposed for predicting the antigenic determinant residues and then a spatial clustering algorithm is adopted to identify the potential epitopes. Firstly, we explore various discriminative features from primary sequences. Secondly, cost-sensitive ensemble scheme is introduced to deal with imbalanced learning problem. Thirdly, we adopt spatial algorithm to tell which residues may potentially form the epitopes. Based on the strategies mentioned above, a new predictor, called CBEP (conformational B-cell epitopes prediction), is proposed in this study. CBEP achieves good prediction performance with the mean AUC scores (AUCs) of 0.721 and 0.703 on two benchmark datasets (bound and unbound) using the leave-one-out cross-validation (LOOCV). When compared with previous prediction tools, CBEP produces higher sensitivity and comparable specificity values. A web server named CBEP which implements the proposed method is available for academic use.

## 1. Introduction

Epitopes or antigenic determinants are the components of antigen membrane receptors which irritate specific interaction with special antibodies [1]. B-cell epitopes are those of spatially proximate residues in antigens which can be recognized and bounded by certain antibodies. Experimental recognition of B-cell epitopes is time-consuming and resource intensive. Therefore, it will be helpful to explore effective computational approaches for reliably identifying the B-cell epitopes in antigens.

Due to the significance of B-cell epitopes in prophylactic and therapeutic biomedical applications [2], various approaches have been proposed in epitope prediction and obtained some achievements [3–19]. B-cell epitopes are of two categories: linear epitopes and conformational epitopes. Since the pioneering work of Hopp [3] on the linear B-cell epitope prediction, many methods [4–8] have been proposed to predict linear epitopes by using residue propensities, that is, hydrophilicity, flexibility, and solvent accessibility. Although the proportion of linear epitopes is very small while the proportion of conformational epitopes is ~90%, the study on conformational epitopes began very late on account of its difficulty. In 2005, CEP [9] was the first study which used solvent accessibility to predict conformational epitopes. DiscoTope [10] predicted antigenic determinants based on antigen 3D structures. The predicted scores were obtained by combining the propensity scores of residues and the contact numbers. SEPPA [11] was another structure-based predictor, which produced a propensity score for a target residue by considering its adjacent residues' information. PEPITO [12] was proposed by feeding linear regression with residue properties and half sphere exposure values. EPSVR [13] built a support vector regression model with epitope propensity scores and some other epitope discriminative features. EPMeta [13] was a metamethod which combined the predicted results from existing web tools to produce the final results. In [14], Zhang et al. introduced the “thick surface patch” to consider the impact of internal residues to the surface residues. Note that almost all abovementioned methods predicted the antigenic residues as belonging to one single epitope without considering multiple nonoverlapping epitopes for an antigen. Considering this, Zhao et al. [15] divided an antigen surface graph into subgraghs by using a Markov clustering approach and then distinguished these subgraphs as epitopes or nonepitope subgraphs. Instead of making predictions based on structures, which need essential 3D structure information, some recent studies explored epitopes based on simple sequence information. In 2010, CBTOPE [16] made the first attempt on predicting conformational epitope from antigen sequences. BEST [17] was a sequence-based predictor that utilized a two-stage design. SVMTrip [7] combined the similarity and occurring-frequency distribution of tripeptides to predict epitopes. BEEPro [8] adopted a linear averaging scheme on 16 properties to recognize both linear and conformational epitopes. As the epitopes prediction was an imbalanced problem, Zhang et al. [18] built an ensemble model using bootstrap technique to deal with imbalanced datasets. Another ensemble method from Zheng et al. [19] was published recently using AdaBoost and the resample method to improve prediction performance.

Although much progress has been made in computational approaches for B-cell epitope prediction, there still exist several aspects for further investigation.

Firstly, many structure-based approaches require 3D structure information as inputs to build prediction models. These methods are invalid when no homology templates can be found for the target antigen protein. Therefore, in this paper, our aim is to develop a powerful predictor for the identification of conformational B-cell epitopes using template-free (sequence-based) approach. Several sequence-derived features are explored to distinguish the epitopes from nonepitopes. These features include evolutionary profile, secondary structure, disorder zone, dipeptide composition, and physicochemical properties.

Secondly, it is apparent that B-cell epitopes prediction is a typical imbalanced learning problem, for which the number of positive samples (epitopes) is much smaller than that of negative samples (nonepitopes). Traditional statistical machine learning algorithms, which tend to ignore the rare samples, often lead to the invalid predicted results under these circumstances. Reported solutions for dealing with imbalanced dataset can be classified into data level and algorithm level approaches [20]. At the data level, the purpose is to rebalance the dataset, such as undersampling technique and oversampling technique [21]. At the algorithm level, the purpose is to search for a proper bias towards the rare samples, such as recognition-based algorithm and cost-sensitive algorithm [22–24]. In this study, cost-sensitive boosting algorithm is firstly introduced for solving serious imbalance samples and building prediction models. The results on two benchmark datasets show that this approach successfully predicts the antigenic determinant residues and outperforms many existing approaches.

Finally, a common drawback of most existing sequence-based B-cell epitope prediction methods is that they are residues-state prediction; that is, they can only simply predict the antigenic determinant residues from sequences but cannot tell which residues may potentially form the real epitopes. Commonly, linear epitopes consist of continuous residues in sequences, while conformational epitopes consist of residues discontinuous in the sequences but spatial proximal [18]. This arouses the consideration of whether spatial clustering algorithm with proper threshold can obtain better results on potential conformational B-cell epitopes prediction. Here, we will computationally investigate the level of residues aggregation in spatial space and try to adopt spatial clustering algorithm in this field.

Based on the strategies mentioned above, a novel method CBEP was proposed for identifying conformational B-cell epitopes by adopting cost-sensitive ensemble scheme with the combination of sequence-derived features and a spatial clustering algorithm for predicting potential epitopes.

## 2. Materials and Methods

### 2.1. Data Collection

In order to reach a consensus assessment with previous methods [9–13, 25, 26], two benchmark datasets including bound structure dataset and unbound structure dataset, which complied from the Rubinstein's bound structure dataset [18, 24, 27] and Liang's unbound structure dataset [13, 18, 25], are also used in this paper.

In addition, to compare our method with previous prediction tools, Liang's [13] 19 antigen structures and sequences with annotated real epitopes are served as independent dataset in this study. The structures of Liang's independent dataset are used to evaluate the structure-based tools, while the primary sequences are used to assess the sequence-based tools.  Table 1 summarizes the detailed compositions of abovementioned three datasets.

### 2.2. Feature Construction

In this study, evolutionary profile, secondary structure, disorder zone, dipeptide composition, and physicochemical properties are combined to form feature vectors for the machine learning techniques. All features are described as follows.

#### 2.2.1. Evolutionary Profile

Here, evolutionary profile is obtained from position specific scoring matrix (PSSM). The PSSM is generated by the program “blastpgp” [28] to search the Swiss-Prot database (released on 15 May, 2011) using default parameters (3 iterations (−*j* 3) and *e*-value threshold 0.001 (−*h* 0.001)) for multiple sequence alignment against the query sequence. For a protein chain with *L* residues, the PSSM is composed of 20 × *L* moments. The obtained PSSM scores are normalized to the interval [0, 1] by the logistic function:

(1) $$\begin{array}{c} f\left(x\right)=\frac{1}{1+e^{-x}}. \end{array}$$

A sliding window of *N* neighboring residues is used to represent the evolutionary profile of a sequence. (*N* − 1)/2 pseudoterminal residues are, respectively, added in the beginning and the end of each sequence. For example, when the window is 9, we add 4 pseudoterminal residues in the head and the tail of the sequence. For the pseudoterminal residue, the values of evolutionary profile features are 0. Finally, each protein residue is represented by 20 × *N* features.

#### 2.2.2. Secondary Structure

This paper includes secondary structure that came from the differences in distributions of the residue depth, which have a strong impact on the epitopes distributions on the protein surface [29]. PSIPRED [30] applied two-stage neural networks to predict secondary structures based on PSIBLAST. The result of PSIPRED is encoded in three lists predicted possibilities for each residue being helix, coil, and strand. The secondary structure features are composed of 3 × *N* features that concern probabilities in a window of *N* adjacent residues using exactly the same scheme as mentioned above.

#### 2.2.3. Disorder Zone

Unstructured regions or natively disordered zone is proved to be closely related with molecular assembly, molecular recognition, surface solvent accessibility, and protein interaction [31–33]. Thus, they are supposed to be useful for protein structure and function predictions. In this work, DISOPRED [34] are used to output the predicted disorder status for each residue in the sequences. As a result, a residue is represented by a 1 × *N* feature which concern statuses in a window of *N* adjacent residues being ordered or disordered.

#### 2.2.4. Dipeptide Composition

The dipeptide is found widely used in proteins and protein-related systems [35–38]. The dipeptide composition for a given protein sequence consists of 420 components. The first 20 components are the traditional amino acid composition (AAC), followed by 400 dipeptides, that is, AA, AC⁡,…, YW, YY; the 400 dipeptides are calculated using the following equation:

(2) $$\begin{array}{c} {\text{content}}_{\text{dep}(i)}=\frac{\sum \text{dep}\left(i\right)}{\sum \text{dipeptides}}, \end{array}$$

where dep(*i*) represents the *i*th dipeptide of the 400 dipeptides, *i* = 1, 2,…, 400.

#### 2.2.5. Physicochemical Properties

Many studies [4–6, 12–18, 39] pointed out that the physicochemical properties of residues were closely associated with the locations of conformation B-cell epitopes. These physicochemical properties include hydrophilicity [14], flexibility [5], accessibility [40], polarity [41], exposed surface [42], and turns [43]. For each residue, the physicochemical features are encoded by a 3 × *N*-dimensional vector that concerns physicochemical properties in a window of *N* adjacent residues.

### 2.3. Fisher-Morkov Selector and Incremental Feature Selection

Empirically speaking, the combination of various types of features should lead to better prediction performance than the individual features. However, information redundancy brought by some features may lead to an unwanted poor performance. To solve this problem, we adopt Fisher-Markov selector [44] to search for optimal feature subset from high-dimensional feature space. Fisher-Markov selector is proved to be a successful method to select those features which can describe the intrinsic differences among the possible classes. In this algorithm, Markov random field optimization schemes are used to solve the formulated objective functions for choosing the best features. After computing the coefficient values using the selector, the ranked feature list will be obtained. Then, incremental feature selection (IFS) procedure is adopted to select the optimal feature set. Each feature in the feature list is added one by one from the head of the list to the tail. When a new feature is added, a new feature subset is generated. *N* different feature subsets will be obtained for the total *N* features. The *i*th feature set can be formulated as

(3) $$\begin{array}{c} \text{Se}{\text{t}}_{i}=\left\{\text{featur}{\text{e}}_{1},\text{featur}{\text{e}}_{2},\ldots ,\text{featur}{\text{e}}_{i}\right\}\;\left(1<i<N\right). \end{array}$$

For each feature subset, a predictor is built and tested using LOOCV on the training dataset. As a result, *N* predictors will be built for the *N* feature subsets. After obtaining the accurate rates of *N* predictors, an IFS scatter plot will be drawn to identify the optimal feature subset (see Section 3.2).

### 2.4. Cost-Sensitive Boosting Method for Antigenic Determinant Residues Prediction

Conformational B-cell antigenic determinant residues prediction is a typical imbalanced classification problem; that is, the number of antigenic determinant residues and nonantigenic determinant residues differs significantly. Most traditional machine learning algorithms are designed to reduce both training and generalization errors and tend to pay less attention to the rare cases. Thus, they generally perform poor performance on the imbalanced datasets. To circumvent this problem, boosting algorithm is adopted to improve the classification performance for the imbalanced dataset. Most boosting algorithms iteratively change the weight distribution of the data space, construct weak classifiers, and boost them to a final strong classifier. When a new weak classifier is added, the samples will be reweighted with correctly classified examples losing weight and misclassified examples gaining weight. The objective of boosting algorithm is to develop a classifier team *H*(*x*) = {*h*_1_, *h*_2_,…, *h*_*k*_} by focusing on those misclassified samples in the previous rounds of learning [21]. The base classifier *h*_*i*_ that joins the ensemble at step *i* is trained on a training subset which is randomly sampled from the training dataset.

The performance of conventional machine learning algorithms is based on their accuracy of classifying positive samples from negative samples. Nevertheless, accuracy may not be the only evaluation criterion in which rare class may be more valuable to be recognized. Compared with traditional machine learning algorithm, which treats samples of different classes equally, cost-sensitive algorithm associates a cost-value with each sample to denote the different importance for identification. Here, a cost matrix is used to encode the different cost of each type of misclassification (Table 2). Let Cost(*i*, *j*) denote the penalty of identifying a sample from class *i* as class *j*. Thus, Cost(+, −) is the cost of misidentifying a positive sample as a negative one, while Cost(−, +) indicates the opposite case. For the classical two class problem, the positive class is the rare class with higher recognition importance, while the negative class is the majority class with less recognition importance. Therefore, in B-cell antigenic determinant residues prediction, the penalty of misidentifying an antigenic determinant residue outweighs that of misidentifying a nonantigenic determinant residue (i.e., Cost(+, −) > Cost(−, +)); making an accurate identification usually indicates zero penalty (i.e., Cost(+, +) = Cost(−, −) = 0). The higher the value is, the more the importance of recognizing this sample is. In summary, the cost-sensitive algorithm is used to minimize the total misclassification cost by considering the various costs of different misclassification types.

Here, we incorporate cost items into the architecture of boosting algorithm to mark different values of various samples (rare antigenic determinant residues and prevalent nonantigenic determinant residues). Let {(*l*_1_, *x*_1_, *c*_1_), (*l*_2_, *x*_2_, *c*_2_),…, (*l*_*m*_, *x*_*m*_, *c*_*m*_)} be a list of training samples, where *l*_*i*_ ∈ {−1, +1} is the class label; *x*_*i*_ is the feature vector; and *C*_*i*_ ⊂ [0, +*∞*) is a cost item marked on each sample. Given a sample, each subclassifier will produce a predicted score. The final predicted score will be obtained by firstly normalizing each score using *Z*-score function and then transforming that using tanh function. Detailed pseudocode for cost-sensitive boosting scheme is given as in Procedure 1.

### 2.5. Clustering Antigenic Determinant Residues to Epitopes

**Figure 1:**
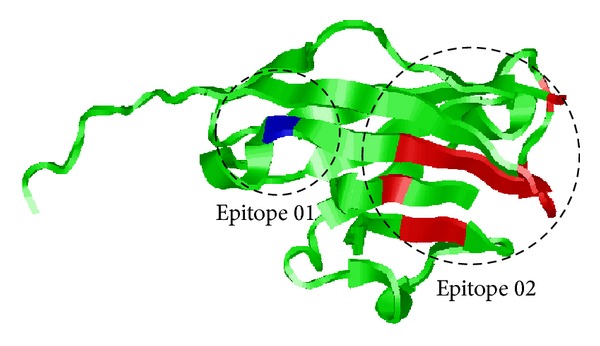
Visualization of two epitopes for chain A of antigen myelin oligodendrocyte glycoprotein (1PKO).

Up to now, all existing sequence-based conformational B-cell epitope predictors can only perform antigenic determinant residue state prediction; that is, they can only predict antigenic determinant residues rather than real epitopes. However, in practical applications, it will be more valuable and meaningful if the predictor can point out which antigenic determinant residue(s) can potentially form an epitope. Previous studies [45, 46] have pointed out that antigenic determinant residues located in antigen-antibody complex tend to cluster in the space. Taking myelin oligodendrocyte glycoprotein (PDBID: 1PKO) as an example, we drew its 3D structure with cartoon representation (Figure 1). As shown in the figure, the area colored blue and red is antigenic determinant residues and has been spatial clustered to form epitopes 01 and 02.

Based on previous researches and observations, here, a postprocessing procedure is developed to further investigate which of the antigenic determinant residues being predicted in previous processes may actually form an epitope.

Detailed pseudocode for spatial clustering scheme is given as in Procedure 2.

**Figure 2:**
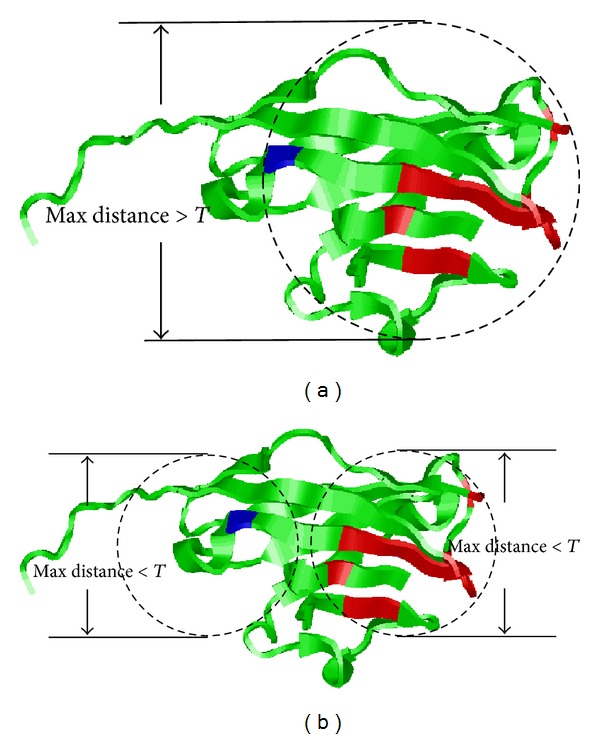
Visualization of spatial clustering procedure on the predicted antigenic determinant residues for 1PKO. (a) Cluster all the predicted antigenic determinant residues in one cluster; (b) split the cluster into two smaller clusters based on predefined threshold.

**Figure 3:**
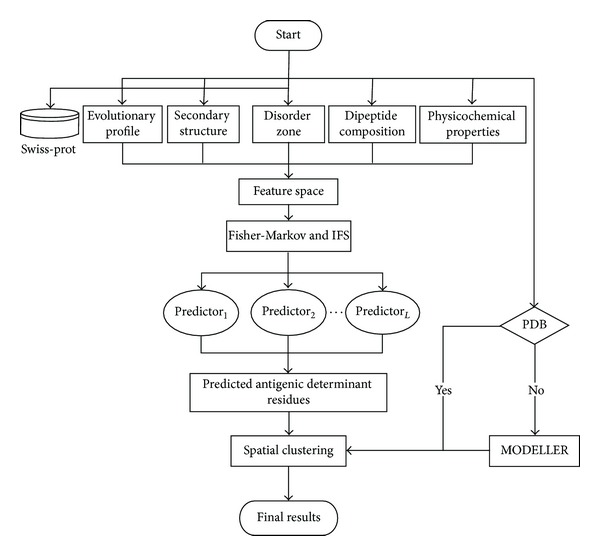
The system architecture of the proposed prediction model.

In the spatial clustering algorithm, the only, but crucial, parameter is the clustering threshold *T* which determines how many epitopes will be obtained in the end. Obviously, a small threshold often produces large number of clusters, while a large threshold often leads to small number of clusters. Thus, it is vital to set an appropriate threshold for the spatial clustering algorithm. Here, we follow the previous study [47] to solve this problem. First of all, *R*_avg is calculated to represent the average distance between the antigenic determinant residues and the centers of their corresponding epitopes. In both bound and unbound datasets, the *R*_avg is about 19 ± 2 Å. Then, the threshold *T* can be initialized as *T* = *α* · (2 × *R*_avg), where *α* is a coefficient which adjusts the distance between two epitopes. After empirically testing *α* based on bound and unbound dataset, the best clustering performance was obtained when *α* = 1.1 (*T* = 41.8 ± 2.4 Å). Using abovementioned algorithm, the antigenic determinant residues of 1PKO are spatially clustered into two clusters (Figure 2). The system architecture of the proposed model is illustrated in Figure 3.

### 2.6. Assessment of Prediction Accuracy

The performance of the proposed model is evaluated by the LOOCV. For comparison with other methods, the performance of this study is measured by several metrics: accuracy (ACC), sensitivity (SN), specificity (SP), *F*-measure (*F*), and the area under receiver operating characteristic (ROC) curve (AUC). Consider

(4) $$\begin{array}{c} \text{ACC}=\frac{\text{TP}+\text{TN}}{\text{TP}+\text{TN}+\text{FP}+\text{FN}},\\ \text{SN}=\frac{\text{TP}}{\text{TP}+\text{FN}},\\ \text{SP}=\frac{\text{TN}}{\text{TN}+\text{FP}},\\ F=2\times \frac{\text{TP}}{2\times \text{TP}+\text{FN}+\text{FP}}, \end{array}$$

where TP and FN stand for the correctly and incorrectly predicted antigenic determinant residues and TN and FN represent the correctly and incorrectly predicted nonantigenic determinant residues. The ROC curve is to plot the true positive rate against false positive rate, and the AUC is a reliable measure for evaluating classifier performance. In the paper, the AUC is the key criteria for assessing the optimal classifier.

## 3. Results and Discussion

### 3.1. Features Analysis and Optimal Window Selection

In order to assess the impact of various window length which is shifted over antigen features, four individual feature-based models (evolutionary information, secondary structure, disorder zone, and physicochemical properties) are constructed on two benchmark datasets (bound and unbound). The performances of various models are presented in Tables 3 and 4, respectively.

As shown in Tables 3 and 4, four types of features all have the abilities of differentiating antigenic determinant residues from nonantigenic determinant residues. Specifically, the performance varies with different window length. Generally speaking, for the bound dataset, the 11-residue window models perform best among all individual feature-based models. For the unbound dataset, the performance of models with 11-residue window is close to that with 13-residue window. For convenience, the 11-residue window is chosen in this study.

We also assess the performance of prediction model constructed by dipeptide composition. Evaluated by LOOCV, the model achieves the AUCs of 0.633 and 0.618 for the bound and unbound dataset, respectively.

As mentioned above, all five sequence-derived features make contribution to differentiate antigenic determinant residues from nonantigenic determinant residues. Therefore, a total of 750 (750 = 20 × 11 + 3 × 11 + 1 × 11 + 6 × 11 + 420) features can be obtained to represent a residue. Shown in Table 5 is a breakdown of the total 750 features for a residue of an antigen by considering its sequence-based information and physicochemical properties.

### 3.2. Results of Fisher-Markov Selector and Incremental Feature Selection

**Figure 4:**
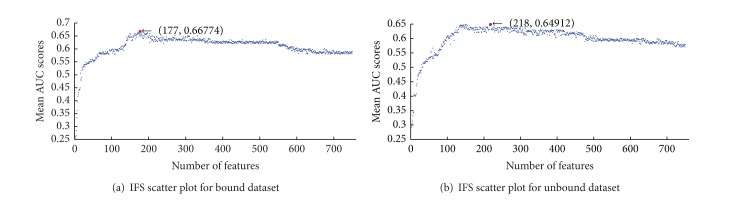
The IFS scatter plots of 750 features for bound dataset (a) and unbound dataset (b).

Based on the scores of Fisher-Markov, individual classifiers were built recursively by adding features from the head of the scores list to the tail one by one. Each subclassifier from cost-sensitive boosting classifiers will produce an AUC. The mean AUCs are calculated to represent the performance for each feature subsets. As shown in Figures 4(a) and 4(b), the mean AUCs reach their maximum when 177 and 218 features are selected for bound and unbound dataset, respectively.

**Figure 5:**
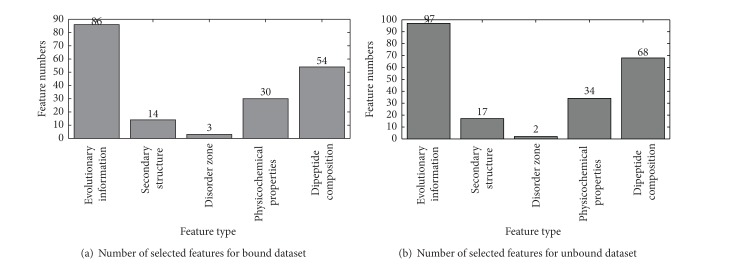
The distribution of each feature type in the final optimal feature subset for bound dataset (a) and unbound dataset (b).

To discover the contribution of each feature type, we further investigate the distribution of different feature types in the final optimal feature subset (Figures 5(a) and 5(b)). It shows that PSSM plays important roles in differentiating antigenic determinant residues from nonantigenic determinant residues. Evolution is an eternal process which impenetrates the whole history of life. The evolution of protein sequences involves the changes, insertions, and deletions of single residue or peptide along with the entire development of proteins [48]. Although some similarities were gradually eliminated after a long time, the corresponding zone having the same biological function may still share some intrinsic attributes [49]. This explains why PSSM occupies a very big part of the optimal subset.

**Figure 6:**
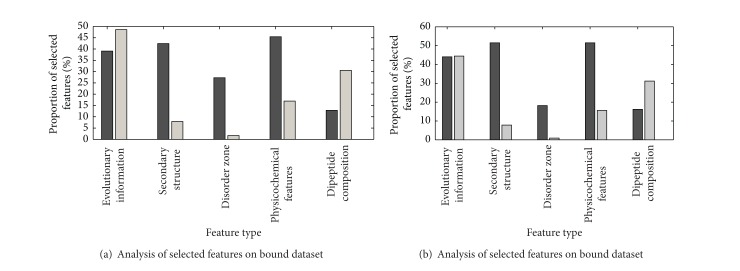
The proportion of each type of features in the final optimal feature subset for bound dataset (a) and unbound dataset (b). The black bars represent the percentage of the selected features accounting for corresponding feature type, and the gray bars represent the percentage of the selected features accounting for the whole optimal feature subsets.

We also calculate different types of features accounting for the various proportions of the optimal feature subset, as can be seen in Figures 6(a) and 6(b). The black bars represent the percentage of the selected features accounting for corresponding feature type, and the gray bars represent the percentage of the selected features accounting for the whole optimal feature subsets. Although within the final optimal feature subset few disorder features are selected, we cannot say that disorder features are not tightly related to antigenic determinant residues. Among the eleven disorder features, three and two features are selected in the optimal feature subsets for two benchmark datasets.

### 3.3. Why We Choose SVM as the Final Classifier?

In addition to SVM, ANN and KNN are also widely used in pattern recognition. For comparison, ANN and KNN are used to build prediction models as well (ANN is implemented by Weka [50], while KNN and SVM are implemented by MATLAB). As shown in Table 6, SVM-based model gives out better predicted performance than ANN-based and KNN-based models with default parameters. What is more, the structure of ANN is more complex than SVM and KNN, and KNN is sensitive to the value of *K*. Therefore, SVM is used in this study as a classification engine. The parameters for bound-dataset-based model are *C* = 32, *γ* = 0.003022 using radial basis function and for unbound-dataset-based model are *C* = 8, *γ* = 0.000068 using Gaussian kernel function.

### 3.4. The Performance of Cost-Sensitive Ensemble Models

**Figure 7:**
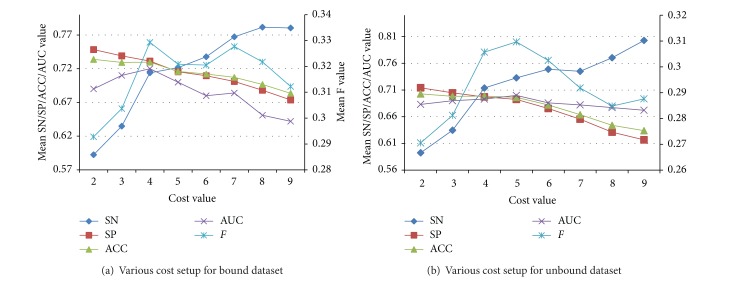
Different performance of various cost setups from 2 to 9. For bound dataset, when cost value is set as 4, *F*-measure and AUC reach the peak values of 0.3302 and 0.721, respectively (a). For unbound dataset, the *F*-measure and AUC reach the maximal values of 0.3117 and 0.703 with cost being set as Figure 5(b).

To deal with the imbalanced samples, we adopt cost-sensitive ensemble technique to build the prediction models. Given an original dataset, the cost setup is usually unknown in advance. A higher cost setup for the rare samples than that for the prevalent samples means that more weights will be boosted on the rare samples. However, some “noisy” data will be included inevitability. Therefore, it is significant to determine the cost value. In this study, various cost values (positive sample (epitopes) versus negative samples (nonepitopes)) are tested from 2 to 9. As shown in Figure 7, with the increase of the cost value on the rare positive samples versus negative samples (epitopes versus nonepitopes), more weighted positive samples are boosted to improve prediction performance and more relevant epitopes tend to be identified. However, with the increase of cost value, the ability of nonepitopes learning decreases simultaneously. Here, *F*-measure and AUC values are used to adapt the balance of learning from both negative and positive samples. For bound dataset, when cost value is set as 4, *F*-measure and AUC reach the peak values of 0.3302 and 0.721, respectively (Figure 7(a)). For unbound dataset, the *F*-measure and AUC reach the maximal values of 0.3117 and 0.703 with cost being set as 5 (Figure 7(b)). Finally, 4 and 5 are chosen as the cost values in bound-dataset-based model and unbound-dataset-based model, respectively.

### 3.5. Comparison with Other Ensemble Methods

To further assess the performance of cost-sensitive ensemble algorithm, four ensemble algorithms, namely, Direct Combination, EasyEnsemble [51], BalanceCascade [51], and SMOTEBoost [52], are adopted in this paper using the optimal feature subsets. EasyEnsemble independently samples several subsets from the majority class. For each subset, a classifier is built using the subset and minority samples. Finally, all subclassifiers are combined to form an ensemble classifier. BalanceCascade is similar to EasyEnsemble except that it removes correctly classified majority class examples of trained classifiers. Instead of using undersampling strategy, SMOTEBoost combines the synthetic minority oversampling technique and boosting strategy to deal with the imbalanced dataset.  Table 7 lists the performance comparison of different ensemble methods on the two benchmark datasets. From Table 7, it is clearly found that cost-sensitive ensemble algorithm produces the best performance with the highest *F* values as well as AUCs. Note that, although direct combination gives out the highest SP values and ACCs, it is indeed invalid in epitope prediction for it fails to identify the rare samples (antigenic determinant residues). More importantly, Table 7 also figures that cost-sensitive ensemble strategy performs better than data-level algorithms in epitopes prediction.

### 3.6. Comparison with Other Epitopes Prediction Methods

In this section, a number of recently published approaches for predicting conformational B-cell epitopes are used for comparison with our new proposed method. These approaches include CEP [9], DiscoTope [10], SEPPA [11], PEPITO [12], EPSVR [13], EPMeta [13], Epitopia [25], EPCES [53], and ElliPro [26]. These approaches can be classified into two types according to the datasets for model building. CEP, Ellipro, SEPPA, PEPITO, DiscoTope, and Epitopia are constructed for identifying B-cell epitopes from bound dataset, while the rest are designed for identifying B-cell epitopes from unbound dataset.

Firstly, we compare our method with the recent bound-structures-based approaches on the bound dataset using LOOCV. DisoTope and Epitopia produce the mean AUCs of 0.60 and 0.59, and BPredictor yields the mean AUCs of 0.633 [18]. Zhang's work gives out the mean AUCs of 0.687 [18]. Zheng's work achieves the mean AUCs of 0.672 using 5-fold cross-validation [19]. Here, our model produces the mean AUCs of 0.721.

When compared with unbound-dataset-based predictors, our method obtains the best AUCs of 0.703 using the same evaluation measure. EPSVR, EPCES, and BPredictor achieve the mean AUCs of 0.670, 0.644, and 0.654, respectively [18]. Zhang's work yields the mean AUCs of 0.651 [18]. Zheng's work gives out the mean AUCs of 0.642 using 5-fold cross-validation [19].

In addition, Liang' independent dataset [13] is used to compare our approach and previous methods. The mean AUCs of DiscoTope, SEPPA, EPITOPIA, BPredictor, EPCES, EPSVR, and CBTOPE calculated by their servers are 0.579, 0.589, 0.572, 0.587, 0.569, 0.606, and 0.607 [18]. Zhang's work gives out the mean AUCs of 0.600 and 0.601 on bound-dataset-based and unbound-dataset-based models, respectively. Our models are firstly built on the bound and unbound dataset, and then the two models are tested by Liang's independent dataset. Finally, our approach gives out the mean AUCs of 0.645 and 0.637, respectively. Although our predictor achieves the best performance, the difference between our predictor and other predictors is not statistically significant by using pairwise Student's* t*-test, partly due to the small number of Liang's independent datasets [13, 14].

In [18], Zhang et al. firstly introduced ensemble scheme into predicting antigenic determinant residues. Zheng et al. [19] adopted the AdaBoost algorithm and resample method to deal with the imbalanced dataset. Compared with conventional ensemble scheme, we add cost-sensitive strategy into boosting scheme. Trained on the same benchmark datasets, our models produce obviously better performance for the independent dataset. Therefore, the cost-sensitive ensemble algorithm that incorporates cost-sensitive scheme produces more robust performance than conventional ensemble simply combining multiple predictors for predicting.

Compared with other predictors, our models produce higher SN and comparable SP on bound, unbound, and independent datasets. Note that cost-sensitive boosting scheme is introduced in this paper to identify the rare epitope class (positive class); more positive samples tend to be identified, so the SN arises obviously; nevertheless, some negative samples may be misidentified as well and the SP gives a slight promotion.

### 3.7. Performance of Spatial Clustering

Two new invited measures [48] (*V*_site_, *V*_*p*_) are adopted here to assess the performance of proposed spatial clustering algorithm adopted on the previous predicted antigenic determinant residues. Consider

(5) $$\begin{array}{c} V_{\text{site}}=\frac{\sum \text{epitop}{\text{e}}_{\text{pre=obs}}}{\sum \text{epitop}{\text{e}}_{\text{pre}}},\\ V_{p}=\frac{\sum \text{antige}{\text{n}}_{\text{pre=obs}}}{\sum \text{antigen}}, \end{array}$$

where ∑epitope_pre=obs_ indicates the number of correctly predicted epitopes; ∑epitope_pre_ indicates the sum of all predicted epitopes; ∑antigen_pre=obs_ represents the number of correctly predicted antigens; and ∑antigen is the sum of all antigens. In this paper, an observed epitope is regarded as a correctly predicted one if more than 30% of its antigenic determinant residues are included in the predicted epitope; an antigen is considered being correctly predicted if all the epitopes are successfully identified and the number of the identified epitopes is equal to that of the observed epitopes. For the bound dataset, the values of *V*_site_ and *V*_*p*_ are 53% and 55%. For the unbound dataset, the values of *V*_site_ and *V*_*p*_ are 52% and 53%. To evaluate the proposed clustering algorithm, two popular spatial clustering algorithms, namely, partitioning around medoids (PAM) [54] and CLARANS [55], are adopted here to predict epitopes clusters. In this study, PAM and CLARANS are implemented by R and MATLAB, respectively; the optimal performance and corresponding parameters are given in Table 8. Experimental results showed that our algorithm could indeed result in better performance. It seems that the results have space to be improved, but they are still encouraging as they are obtained from simple primary sequence. Actually, this is the first study that introduces spatial clustering algorithm to conformational B-cell epitopes prediction. More effective methods are needed to explore this field. It is expected to be particularly useful when no template can be found for a given antigen. In this situation, cost-sensitive ensemble predictors are firstly used to predict the antigenic determinant residues, and then spatial algorithm is performed on a modeled structure from the algorithms like MODELLER and so forth to predict the potential epitopes.

### 3.8. Implementation of CBEP

**Figure 8:**
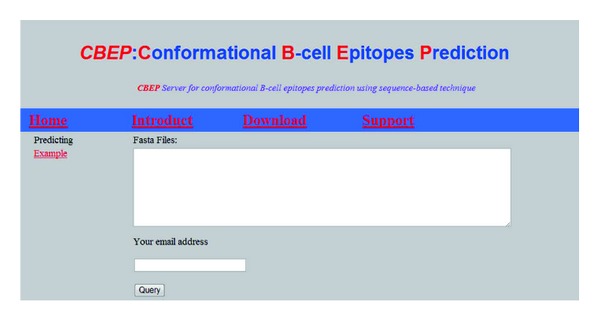
The home page of CBEP server.

For the convenience of biology scientists, CBEP has been implemented as a free web server on Linux platform. A brief guide is given below to describe how to use it. (i) Access the web server at http://59.73.198.144:8088/CBEP/ and* Home* is the default interface displayed (Figure 8). Click on the* Introduction* link to see a detailed description about the server. (ii) Either type or paste the antigen sequence (or list of sequences) into the text box. Note that, the input sequence should be in the FASTA format, which consists of a single-line description and lines of sequence information. Click on the* Example* link to see the example sequence. You will also be asked to type your email address. The predicting results will be sent to you as soon as the computational process is finished. (iii) Click on the* Query* button to submit the computation request. Generally speaking, it takes no more than 2 minutes to predict the antigenic determinant residues for a sequence with no more than 300 amino acids.

## 4. Conclusions

In this paper, we proposed a novel B-cell epitope predictor CBEP. The antigen protein sequences are firstly encoded with various sequence-derived features; then cost-sensitive ensemble scheme is adopted to predict the antigenic determinant residues; finally the predicted antigenic determinant residues are fed into the spatial clustering algorithm to evaluate the potential B-cell epitopes. Experiment results on bound datasets, unbound datasets, and independent datasets have demonstrated the efficacy of the proposed model. In addition, our model could predict potential epitopes from antigenic determinant residues with a spatial clustering process. It is an enlightening attempt. Our future works will focus on improving the prediction accuracy by developing more powerful classifiers and more accurate spatial clustering algorithms. For the convenience of biology scientists, CBEP has been implemented as a free web server located at http://59.73.198.144:8088/CBEP/.

## Figures and Tables

**Procedure 1:**
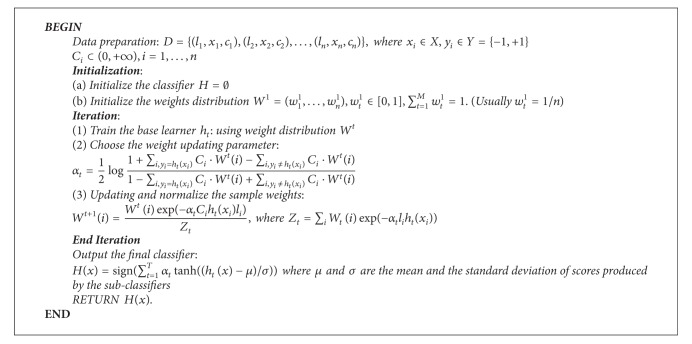
Procedure Cost-Sensitive Boosting scheme.

**Procedure 2:**
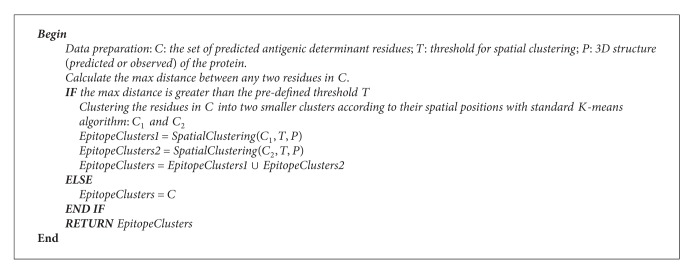
Procedure Spatial clustering algorithm.

**Table 1:** Detailed compositions of the bound, unbound and independent datasets.

Dataset	No. of Sequences	(numP, numN)∗
Bound structure	83	(1076, 16744)
Unbound structure	48	(898, 8759)
Independent	19	(440, 4944)

*(numP, numN) represent the numbers of positive (antigenic determinant residues) and negative (non-antigenic determinant residues) samples, respectively.

**Table 2:** Confusion matrix.

	Actually positive	Actually negative
Predict positive	Cost(+, +)	Cost(+, −)
Predict negative	Cost(−, +)	Cost(−, −)

Cost values are set according to different classification results. Generally, for a rare positive and prevalent negative samples, Cost(+, −) > Cost(−, +). And Cost(+, +) = Cost(−, −) = 0 denotes no penalty for a correctly predicted sample.

**Table 3:** Mean AUCs of proposed models for the bound dataset using LOOCV.

Window	#1	#2	#3	#4	Average
7	0.629	0.633	0.622	0.673	0.629
9	0.644	0.633	0.619	0.675	0.631
11	0.647	**0.640**	**0.625**	0.674	**0.635**
13	**0.648**	0.628	0.611	**0.680**	0.630
15	0.632	0.624	0.614	0.676	0.625

Evolutionary information (#1), secondary structure (#2), disorder zone (#3), physicochemical features (#4).

**Table 4:** Mean AUCs of proposed models for the unbound dataset using LOOCV.

Window	#1	#2	#3	#4	Average
7	0.609	0.623	0.597	0.636	0.616
9	0.611	0.631	0.595	0.639	0.619
11	0.613	**0.636**	0.597	**0.644**	**0.622**
13	**0.615**	0.634	**0.598**	0.641	**0.622**
15	**0.615**	0.630	0.597	0.642	0.621

Evolutionary information (#1), secondary structure (#2), disorder zone (#3), physicochemical features (#4).

**Table 5:** A breakdown of the 750 features.

Feature type	Number of feature	Window size	Total
Evolutionary information	20	11	220
Secondary structure	3	11	33
Disorder zone	1	11	11
Physicochemical features	6	11	66
Dipeptide composition	420	—	420
Summary	—	—	750

**Table 6:** Performance of different machine learning methods, evaluated by LOOCV.

Method	Bound dataset					Unbound dataset				
	*F*	ACC	SN	SP	AUC	*F*	ACC	SN	SP	AUC
ANN	0.294	0.723	0.629	0.732	0.643	0.276	0.678	0.659	0.679	0.645
KNN	0.323	0.744	**0.666**	0.752	0.654	0.298	**0.692**	0.662	0.665	0.648
SVM	**0.330**	**0.750**	0.648	**0.760**	**0.661**	**0.312**	0.689	**0.704**	**0.687**	**0.652**

**Table 7:** Performance of different ensemble methods on bound and unbound datasets.

Method	Bound dataset				Unbound dataset			
	*F*	ACC	SN	SP	*F*	ACC	SN	SP
Direct Combination	0.016	**0.939**	0.008	**0.999**	0.021	**0.907**	0.011	**0.999**
EasyEnsemble	0.168	0.883	0.196	0.927	0.208	0.818	0.256	0.876
BalanceCascade	0.224	0.870	0.314	0.904	0.245	0.808	0.335	0.856
SMOTEBoost	0.252	0.852	0.415	0.879	0.239	0.750	0.423	0.784
Cost-Sensitive	**0.330**	0.750	**0.648**	0.760	**0.312**	0.689	**0.704**	0.687

**Table 8:** Performance of different clustering algorithms on benchmark datasets.

Method	Bound dataset		Unbound dataset	
	*V*_site_	*V*_*p*_	*V*_site_	*V*_*p*_
PAM^1^	41%	46%	40%	41%
CLARANS^2^	45%	48%	48%	51%
Our method^3^	53%	55%	52%	53%

^1^dist = Euclidean distance, *k* = 4; ^2^dist = Euclidean distance, *k* = 3;^ 3^ *R*_avg = 19, *α* = 1.1.

## References

[1] van Regenmortel MH (1989). The concept and operational definition of protein epitopes. *Philosophical Transactions of the Royal Society of London B*.

[2] Irving MB, Pan O, Scott JK (2001). Random-peptide libraries and antigen-fragment libraries for epitope mapping and the development of vaccines and diagnostics. *Current Opinion in Chemical Biology*.

[3] Hopp TP Identification and preparation of epitopes on antigens and allergens on the basis of hydrophilicity.

[4] Parker JMR, Guo D, Hodges RS (1986). New hydrophilicity scale derived from high-performance liquid chromatography peptide retention data: correlation of predicted surface residues with antigenicity and X-ray-derived accessible sites. *Biochemistry*.

[5] Karplus PA, Schulz GE (1985). Prediction of chain flexibility in proteins. A tool for the selection of peptide antigens. *Naturwissenschaften*.

[6] Kolaskar AS, Tongaonkar PC (1990). A semi-empirical method for prediction of antigenic determinants on protein antigens. *FEBS Letters*.

[7] Yao B, Zhang L, Liang S, Zhang C (2012). SVMTriP: a method to predict antigenic epitopes using support vector machine to integrate tri-peptide similarity and propensity. *PLoS ONE*.

[8] Lin SY, Cheng CW, Su EC (2013). Prediction of B-cell epitopes using evolutionary information and propensity scales. *BMC Bioinformatics*.

[9] Kulkarni-Kale U, Bhosle S, Kolaskar AS (2005). CEP: a conformational epitope prediction server. *Nucleic Acids Research*.

[10] Andersen PH, Nielsen M, Lund O (2006). Prediction of residues in discontinuous B-cell epitopes using protein 3D structures. *Protein Science*.

[11] Sun J, Wu D, Xu T (2009). SEPPA: a computational server for spatial epitope prediction of protein antigens. *Nucleic Acids Research*.

[12] Sweredoski MJ, Baldi P (2008). PEPITO: improved discontinuous B-cell epitope prediction using multiple distance thresholds and half sphere exposure. *Bioinformatics*.

[13] Liang S, Zheng D, Standley DM, Yao B, Zacharias M, Zhang C (2010). EPSVR and EPMeta: prediction of antigenic epitopes using support vector regression and multiple server results. *BMC Bioinformatics*.

[14] Zhang W, Xiong Y, Zhao M, Zou H, Ye X, Liu J (2011). Prediction of conformational B-cell epitopes from 3D structures by random forests with a distance-based feature. *BMC Bioinformatics*.

[15] Zhao L, Wong L, Lu L, Hoi SCH, Li J (2012). B-cell epitope prediction through a graph model. *BMC Bioinformatics*.

[16] Ansari HR, Raghava GP (2010). Identification of conformational B-cell Epitopes in an antigen from its primary sequence. *Immunome Research*.

[17] Gao J, Faraggi E, Zhou Y, Ruan J, Kurgan L (2012). BEST: improved prediction of B-cell epitopes from antigen sequences. *PLoS ONE*.

[18] Zhang W, Niu Y, Xiong Y, Zhao M, Yu R, Liu J (2012). Computational prediction of conformational B-cell epitopes from antigen primary structures by ensemble learning. *PLoS ONE*.

[19] Zheng W, Zhang C, Hanlon M, Ruan J, Gao J (2014). An ensemble method for prediction of conformational B-cell epitopes from antigen sequences. *Computational Biology and Chemistry*.

[20] Chawla NV, Japkowicz N, Kotcz A (2004). Editorial: special issue on learning from imbalanced data sets. *ACM Sigkdd Explorations Newsletter*.

[21] Sun Y, Kamel MS, Wong AKC, Wang Y (2007). Cost-sensitive boosting for classification of imbalanced data. *Pattern Recognition*.

[22] Zhou Z-H, Liu X-Y (2010). On multi-class cost-sensitive learning. *Computational Intelligence*.

[23] He H, Garcia EA (2009). Learning from imbalanced data. *IEEE Transactions on Knowledge and Data Engineering*.

[24] Rubinstein ND, Mayrose I, Pupko T (2009). A machine-learning approach for predicting B-cell epitopes. *Molecular Immunology*.

[25] Liang S, Zheng D, Zhang C, Zacharias M (2009). Prediction of antigenic epitopes on protein surfaces by consensus scoring. *BMC Bioinformatics*.

[26] Ponomarenko J, Bui H-H, Li W (2008). ElliPro: a new structure-based tool for the prediction of antibody epitopes. *BMC Bioinformatics*.

[27] Rubinstein ND, Mayrose I, Martz E, Pupko T (2009). Epitopia: a web-server for predicting B-cell epitopes. *BMC Bioinformatics*.

[28] Altschul SF, Madden TL, Schäffer AA (1997). Gapped BLAST and PSI-BLAST: a new generation of protein database search programs. *Nucleic Acids Research*.

[29] Zhang H, Zhang T, Chen K, Shen S, Ruan J, Kurgan L (2008). Sequence based residue depth prediction using evolutionary information and predicted secondary structure. *BMC Bioinformatics*.

[30] McGuffin LJ, Bryson K, Jones DT (2000). The PSIPRED protein structure prediction server. *Bioinformatics*.

[31] Dyson HJ, Wright PE (2005). Intrinsically unstructured proteins and their functions. *Nature Reviews Molecular Cell Biology*.

[32] Haynes C, Oldfield CJ, Ji F (2006). Intrinsic disorder is a common feature of hub proteins from four eukaryotic interactomes. *PLoS Computational Biology*.

[33] Gsponer J, Futschik ME, Teichmann SA, Babu MM (2008). Tight regulation of unstructured proteins: from transcript synthesis to protein degradation. *Science*.

[34] Ward JJ, Sodhi JS, McGuffin LJ, Buxton BF, Jones DT (2004). Prediction and functional analysis of native disorder in proteins from the three kingdoms of life. *Journal of Molecular Biology*.

[35] Chou K-C (2009). Pseudo amino acid composition and its applications in bioinformatics, proteomics and system biology. *Current Proteomics*.

[36] Mohabatkar H (2010). Prediction of cyclin proteins using chou’s pseudo amino acid composition. *Protein and Peptide Letters*.

[37] Chen C, Chen L, Zou X, Cai P (2009). Prediction of protein secondary structure content by using the concept of Chou’s pseudo amino acid composition and support vector machine. *Protein and Peptide Letters*.

[38] Ding H, Luo L, Lin H (2009). Prediction of cell wall lytic enzymes using chou’s amphiphilic pseudo amino acid composition. *Protein and Peptide Letters*.

[39] Saha S, Raghava GPS (2004). BcePred: prediction of continuous B-cell epitopes in antigenic sequences using physico-chemical properties. *Artificial Immune Systems*.

[40] Emini EA, Hughes JV, Perlow DS, Boger J (1985). Induction of hepatitis A virus-neutralizing antibody by a virus-specific synthetic peptide. *Journal of Virology*.

[41] Ponnuswamy PK, Prabhakaran M, Manavalan P (1980). Hydrophobic packing and spatial arrangement of amino acid residues in globular proteins. *Biochimica et Biophysica Acta*.

[42] Janin J, Wodak S, Levitt M, Maigret B (1978). Conformation of amino acid side chains in proteins. *Journal of Molecular Biology*.

[43] Pellequer J-L, Westhof E, van Regenmortel MHV (1993). Correlation between the location of antigenic sites and the prediction of turns in proteins. *Immunology Letters*.

[44] Cheng Q, Zhou H, Cheng J (2011). The fisher-markov selector: fast selecting maximally separable feature subset for multiclass classification with applications to high-dimensional data. *IEEE Transactions on Pattern Analysis and Machine Intelligence*.

[45] Keck Z-Y, Xia J, Wang Y (2012). Human monoclonal antibodies to a novel cluster of conformational epitopes on HCV E2 with resistance to neutralization escape in a genotype 2a isolate. *PLoS Pathogens*.

[46] Stufano A, Capone G, Pesetti B, Polimeno L, Kanduc D (2010). Clustering of rare peptide segments in the HCV immunome. *Self/Nonself*.

[47] Yu D-J, Hu J, Huang Y (2013). TargetATPsite: a template-free method for ATP-binding sites prediction with residue evolution image sparse representation and classifier ensemble. *Journal of Computational Chemistry*.

[48] Chou K-C (1995). The convergence-divergence duality in lectin domains of selectin family and its implications. *FEBS Letters*.

[49] Li B-Q, Hu L-L, Chen L, Feng K-Y, Cai Y-D, Chou K-C (2012). Prediction of protein domain with mRMR feature selection and analysis. *PLoS ONE*.

[50] Hall M, Frank E, Holmes G, Pfahringer B, Reutemann P, Witten IH (2009). The WEKA data mining software: an update. *ACM SIGKDD Explorations Newsletter*.

[51] Liu X-Y, Wu J, Zhou Z-H (2009). Exploratory undersampling for class-imbalance learning. *IEEE Transactions on Systems, Man, and Cybernetics B*.

[52] Chawla NV, Lazarevic A, Hall LO, Bowyer KW SMOTEBoost: improving prediction of the minority class in boosting.

[53] Ponomarenko J, Papangelopoulos N, Zajonc DM, Peters B, Sette A, Bourne PE (2011). IEDB-3D: structural data within the immune epitope database. *Nucleic Acids Research*.

[54] van der Laan MJ, Pollard KS, Bryan J (2003). A new partitioning around medoids algorithm. *Journal of Statistical Computation and Simulation*.

[55] Ng RT, Han J (2002). CLARANS: a method for clustering objects for spatial data mining. *IEEE Transactions on Knowledge and Data Engineering*.

